# Editorial: Innovation in the management of rhinologic disorders

**DOI:** 10.3389/falgy.2026.1930895

**Published:** 2026-08-03

**Authors:** Puya Dehgani-Mobaraki, Juan Maza-Solano, Saad Alsaleh, Gwijde F. J. P. M. Adriaensen, Asiya Kamber Zaidi

**Affiliations:** 1 Department of Otorhinolaryngology, USL Umbria 1, Perugia, Italy; 2 Associazione Naso Sano, Corciano, Italy; 3 Rhinology and Skull Base Unit, Department of Otorhinolaryngology, University Hospital Virgen del Rocío, Seville, Spain; 4 Department of Surgery, University of Seville, Seville, Spain; 5 QUIRONSALUD Sagrado Corazón Hospital, Seville, Spain; 6 Rhinology Study Group of the Young-Otolaryngologists of the International Federations of Oto-rhino-laryngological Societies (YO-IFOS), Paris, France; 7 Security Forces Hospitals Program, General Directorate of Medical Services, Ministry of Interior, Riyadh, Saudi Arabia; 8 Department of Otorhinolaryngology - Head & Neck Surgery, Amsterdam University Medical Center, Amsterdam, Netherlands; 9 Department of Otorhinolaryngology - Head & Neck Surgery, University Hospitals Birmingham NHS Foundation Trust, Birmingham, United Kingdom

**Keywords:** biologics, biomarkers, chronic rhinosinusitis, minimally invasive surgery, nasal polyps, precision medicine, PROMS, rhinology

Rhinologic disorders, including allergic rhinitis (AR), chronic rhinosinusitis (CRS), and chronic rhinosinusitis with nasal polyps (CRSwNP), constitute a significant global health burden, characterized by chronic inflammation, marked heterogeneity, and substantial impact on quality of life. In recent years, advances in immunopathology, molecular biology, and technological innovations have profoundly reshaped both the conceptual understanding and therapeutic landscape of rhinology. This Research Topic, “*Innovation in the Management of Rhinologic Disorders,”* reflects this ongoing transformation, highlighting the transition toward precision medicine, minimally invasive care, and patient-centered management.

A key paradigm shift emerging across the contributions is the movement from phenotype-based classification toward endotype-driven disease stratification. Chronic inflammatory sinonasal diseases are increasingly understood as biologically distinct entities defined by specific immunological pathways. In this context, Vizcarra-Melgar et al. demonstrate that tissue eosinophilia, despite its traditional role as a hallmark of type 2 inflammation, does not correlate with disease control, severity, or recurrence in CRSwNP, whereas peripheral blood eosinophilia shows more consistent associations with clinical outcomes. This observation underscores a critical limitation of single-parameter biomarkers and supports the need for integrated, multidimensional stratification models.

At the molecular level, Kim et al. further refined our understanding of disease pathophysiology by identifying periostin as a central mediator linking type 2 inflammation with tissue remodeling and osteitis. Notably, periostin appears to exert a dual role, contributing not only to osteogenic remodeling but also to the maintenance of extracellular matrix integrity. Their findings reinforce the concept that CRS is not solely an inflammatory condition but also a disease of structural alteration, in which extracellular matrix dynamics play a pivotal role in disease persistence and recurrence.

Expanding beyond the local sinonasal environment, Zhou et al. provide evidence supporting a systemic dimension of rhinologic disease, demonstrating causal relationships between gut microbiota composition, immune traits, and nasal polyp development. These findings align with the emerging concept of a gut–immune–airway axis, suggesting that sinonasal inflammation may be influenced by systemic immunological and microbial factors. This perspective opens new avenues for therapeutic modulation beyond the sinonasal compartment.

Therapeutic innovation is prominently represented by the growing role of targeted biologic therapies. The randomized controlled trial protocols by Toppila-Salmi et al. and Lyly et al. explore acetylsalicylic acid desensitization and anti–IL-5 therapy with mepolizumab, respectively, specifically in patients with severe, uncontrolled CRSwNP associated with NSAID-exacerbated respiratory disease, a subgroup characterized by high disease burden and frequent surgical interventions. These approaches exemplify the integration of immunological profiling into therapeutic decision-making and highlight the importance of individualized treatment strategies in refractory diseases.

In parallel, novel pharmacological strategies are emerging at the interface of nanotechnology and translational medicine. Zhou et al. demonstrate that carbon-based nanomaterials derived from *Zingiberis Rhizoma Carbonisatum* exert anti-inflammatory and immunomodulatory effects through modulation of key signaling pathways, including PI3K/AKT and JAK/STAT. Although currently limited to preclinical models, such approaches suggest that innovative drug delivery systems and bioengineered compounds may complement existing therapies and potentially overcome current treatment limitations.

Surgical innovation remains a cornerstone of rhinologic practice. The review by Alokby highlights the rapid expansion of office-based and minimally invasive procedures, including balloon sinuplasty, cryotherapy, and radiofrequency neurolysis. These techniques reflect a broader shift toward resource-efficient, patient-centered care, enabling effective treatment while reducing the need for operating room infrastructure and general anesthesia. Beyond technical refinement, this evolution represents a reorganization of care delivery consistent with value-based healthcare principles.

The inclusion of severe and life-threatening conditions further emphasizes the breadth of this Research Topic. Liu et al. identify key prognostic factors in invasive fungal rhinosinusitis, including immunosuppression, metabolic dysregulation, and intracranial extension, highlighting the importance of early diagnosis and aggressive multidisciplinary management. This contribution underscores that, despite advances in elective rhinology, complex and rapidly progressive diseases remain a critical clinical challenge.

A particularly relevant dimension of innovation is the integration of patient-reported outcome measures (PROMs) into routine clinical practice. The PROMUSE Respiratory Study by Cherrez-Ojeda et al. provides important real-world data demonstrating that, despite strong guideline recommendations, PROM utilization remains suboptimal. Time constraints and workflow limitations emerge as the primary barriers, even though clinicians widely recognize the value of PROMs in monitoring disease control and evaluating treatment outcomes. These findings highlight a persistent gap between evidence and implementation.

PROMs represent a fundamental component of patient-centered care, capturing dimensions of disease burden—such as quality of life and functional impairment—that are often underestimated in traditional clinical assessment. Their integration into routine practice, particularly through digital platforms and automated systems, has the potential to enhance patient–physician communication and support shared decision-making. However, as demonstrated in this study, successful implementation requires structural, technological, and educational adaptation.

Collectively, the contributions within this Research Topic demonstrate that innovation in rhinology is inherently multidimensional. It encompasses advances in molecular science, therapeutic development, surgical techniques, and healthcare delivery models. Rather than representing isolated advances, these contributions reflect an increasing convergence and integration across these domains. More importantly, it reflects a shift toward integration—between local and systemic disease mechanisms, between technological innovation and clinical application, and between physician-driven and patient-centered care paradigms.

Looking forward, several priorities emerge. The development of composite biomarker frameworks will be essential to operationalize precision medicine. Long-term evaluation of biologic therapies must address not only efficacy but also sustainability and cost-effectiveness. The integration of microbiome research will further expand the conceptual boundaries of rhinology. Standardization of minimally invasive procedures will ensure reproducibility and optimal outcomes. Finally, the widespread implementation of PROMs and digital health tools will be critical to achieving meaningful improvements in patient care. In this context, the integration of multi-omics approaches, and artificial intelligence may further refine disease stratification and therapeutic targeting.

In conclusion, this Research Topic captures a transformative phase in rhinology. The convergence of scientific discovery, technological innovation, and patient-centered approaches is redefining the field. The challenge ahead lies not only in sustaining this momentum but in ensuring that innovation is effectively translated into routine clinical practice, ultimately improving outcomes for patients with rhinologic disorders.

